# Prophylaxis of Ophthalmia Neonatorum: A Request for Statistics

**Published:** 1910-01-08

**Authors:** A. Nimmo Walker

**Affiliations:** 45 Rodney Street, Liverpool


### Editor's Letter-Box.

### PROPHYLAXIS OF OPHTHALMIA NEONATORUM: A REQUEST FOR STATISTICS.

*To the Editor of* The Hospital.

Sir,—I am endeavouring to collect statistics which may throw some light on the vexed question of antisepsis *v.* asepsis in the prevention of ophthalmia in the newly-born. The antiseptic method assumes the possibility of infection in every case, and may be sub-divided into germicidal, where an endeavour is made to kill the germ by strong germicides, such as silver nitrate and corrosive sublimate, and into mechanical, where trust is placed in the removal of the germ by mechanical flushing with feeble antiseptics, such as boracic lotion. The aseptic method, on the other hand, aims at the thorough cleansing of the eyelids before they are opened, and the prevention of inoculation afterwards, nothing being put into the conjunctival sac unless there is definite reason to fear infection.

There are many records of statistics of the antiseptic methods, but as far as I am aware the only statistics of the aseptic method are those quoted by Sydney Stephenson in his Middlemore Prize Essay of 1907. Moreover, the majority of statistics on the subject have been obtained from the lying-in hospitals, and there must be a great store of instructive information in the experiences of general practitioners and of midwives which has never been utilised.

May I ask those of your readers who are interested, for

1. References to recent statistics.
2. Personal experiences in the use of these different methods.

I will be very grateful for any information sent to me at the subjoined address, and for facility of tabulation I beg to suggest that the following table should be used:

| Method Employed | Number of Births | Number of Cases of Inflammation within Four Days of Birth | | Number of Cases of Injured Eyes |
|---|---|---|---|---|
| | | Severe | Slight | |

Details of the bacteriology and probable method of infection will be particularly valuable.

I am obtaining some statistics of this kind from friends in Liverpool, but I venture to ask the hospitality of your columns for this request in order that the scope of the inquiry may be enlarged.

I am, yours faithfully,

A. Nimmo Walker.

45 Rodney Street, Liverpool.

